# Air Pollution and Birth Weight in Connecticut and Massachusetts

**DOI:** 10.1289/ehp.10631

**Published:** 2008-03

**Authors:** Muhammad Towhid Salam

**Affiliations:** Department of Preventive Medicine, University of Southern California, Keck School of Medicine, Los Angeles, California, E-mail: msalam@usc.edu

In their paper, Bell et al. (2007) examined the effects of ambient air pollutants on birth weight in children born in Connecticut and Massachusetts between 1999 and 2002. The study is the largest among the studies conducted in the United States and has provided evidence that, even with pollutant levels that met the air quality standards, significant reductions in birth weight occurred.

The study findings are in line with other articles; however, there are several concerns that need further attention. Infants with preterm birth (born at 32–37 weeks of gestation) were included in the analysis, and this may have affected the overall and the third trimester–specific results. Although these children comprised only 6.7% of the sample, their birth weights were greatly affected (i.e., mean birth weight was 585–1,050 g less than those born at 39–40 weeks of gestation).

In the discussion, Bell et al. (2007) pointed out that the effect of air pollutants on birth weight could be mediated by the effect of these pollutants on preterm birth and/or on fetal growth. It is unclear whether the effects observed in this study were mediated by one or both competing mechanisms. Bell et al.’s Table 6 compared studies that tested pollutant effects mediated only by fetal growth (Basu et al. 2004; Parker et al. 2005; Ritz and Yu 1999; Salam et al. 2005), only by preterm birth (Rogers and Dunlop 2006; Rogers et al. 2000), or by both mechanisms separately (Wilhelm and Ritz 2003, 2005; Woodruff et al. 2003). Bell et al. (2007) could have further benefited readers if they had evaluated pollutant effects on intrauterine growth restriction (IUGR), a metric of pathological fetal growth. Although IUGR is conventionally defined based on the bottom 10th percentile of the birth weight distribution, a better approach would be to define it by < 15th percentile of predicted birth weight based on gestational age, infant sex, and maternal race.

Pollutant data were not available from all counties. As such, different counties contributed to different exposure effects. This makes the comparison between single and two-pollutant models difficult. In Figure 2 (Bell et al. 2007), for example, the effect of particulate matter < 2.5 μm in aerodynamic diameter (PM_2.5_; measured in 13 counties) seems to have been significantly changed when adjusted for carbon monoxide (measured in 7 counties). It would have been more meaningful if sensitivity analyses were conducted comparing one and two-pollutant models restricting to those counties that had data on all exposures.

The detrimental effect of PM_2.5_ on birth weight was significantly larger in black infants than in white infants. Hispanic white infants were at increased risk of low birth weight in a similar population (Maisonet et al. 2001). Thus, combining non-Hispanic and Hispanic whites into one group, it is not possible to determine whether Hispanic infants were disproportionately affected by ambient pollutants. There is significant heterogeneity in the percentages of African-American and Hispanic white population by county (U.S. Census Bureau 2007). For example, within the state of Massachusetts, 25% of residents were African American and about 18% were Hispanic in Suffolk, whereas about 2% each were African American and Hispanic in Barnstable. In addition, in counties with higher proportions of African-American and Hispanic populations, the percentages living in poverty were also much higher. Although Bell et al. (2007) acknowledged within-county heterogeneity, questions remain about whether significant heterogeneity in effects existed across counties.

In summary, the results would have been more appealing if Bell et al. (2007) could provide data to show whether pollutant effects were mediated by preterm birth and/or affected fetal growth, and whether these effects were greater in Hispanics, in mothers who smoked, and in counties with a higher proportion of people living in poverty.
